# Turkish version of the Yale Food Addiction Scale: preliminary results of factorial structure, reliability, and construct validity

**DOI:** 10.1186/s41043-019-0202-4

**Published:** 2019-12-10

**Authors:** Zehra Buyuktuncer, Aslı Akyol, Aylin Ayaz, Reyhan Nergiz-Unal, Burcu Aksoy, Erdal Cosgun, Pınar Ozdemir, Gulden Pekcan, Halit Tanju Besler

**Affiliations:** 1 Department of Nutrition and Dietetics, Faculty of Health Sciences, Hacettepe University, 06230 Ankara, Turkey; 2 Department of Biostatistics and Medical Informatics, Faculty of Medicine, Acıbadem University, Atasehir, Istanbul, Turkey; 3 Department of Biostatistics and Medical Informatics, School of Medicine, Hacettepe University, 06230 Ankara, Turkey

**Keywords:** Food addiction, Yale Food Addiction Scale, Psychometric properties, Validation, Turkish population

## Abstract

**Background:**

Yale Food Addiction Scale (YFAS) was established to identify individuals exhibiting signs of addiction towards certain types of food. This study aimed to develop a Turkish version of the Yale Food Addiction Scale and test its psychometric properties.

**Methods:**

The backward translation techniques were used to develop Turkish versions of the YFAS, and its reproducibility was assessed. Turkish version of the YFAS was administered to a total of 1033 participants (439 men and 594 women), aged 19–65 years. Exploratory factor analysis and confirmatory factor analysis were used to examine the factorial structure of the tool. Construct validity was assessed by principal component factor analysis with varimax rotation. Reliabilities were estimated with Cronbach’s alpha coefficient. The criterion-related validity was tested by the administration of Eating Attitude Test-26 (EAT-26) to all participants.

**Results:**

The primary factor loadings for seven items were ranged between 0.45 and 0.79, and no items cross-loaded onto other factors. The fit indices showed that eight items of the YFAS were a good representation of the item responses and each item loaded significantly on the specified factor (*p* < 0.001 for each). YFAS subscales had a high internal consistency and test–retest reliability. The criterion-related validity of the tool showed a positive relationship with scales of the EAT-26.

**Conclusion:**

Current study suggested that the Turkish version of the YFAS is a reliable, valid, and useful tool for assessing the signs of food addiction in a non-clinical sample.

## Background

The prevalence of obesity and overweight continues to increase dramatically [1]. It has been estimated that 60% of men and 50% of women could be clinically obese by the year 2050 [2]. The determinative effect of environmental factors such as eating habits and nutrition on the development of obesity has been well established [3–5]. However, recent reports suggest that lifestyle-related prevention and treatment strategies do not result in complete success over the long term [6, 7]. One of the main reasons for this outcome has been attributed to individual food preferences [8, 9]. According to this theory, palatable foods that are rich in sugar, fat, and energy may create an addictive response and lead individuals to consume more [10]. Thus, lifestyle modifications toward healthy choices become difficult to maintain. A limited number of studies indicated that food addiction prevalence was found to be high in individuals who were obese and had binge-eating disorders [11–14]. This may explain the relationship between food addiction and a continuous drive to eat in obesity [15].

A biological basis for food addiction has been shown in several studies [16–18]. Interestingly, these studies revealed that food addiction mechanisms exhibited pathways similar to those seen in drug addiction [19]. Hedonic nutrition, which can be described as consumption of food principally because of its palatability, rather than its nutritional value, was shown to be associated with the food reward system in the brain [20]. Mounting evidence substantiates that neurotransmitters such as dopamine, opioid peptides, and related pathways play important roles in hedonic nutrition and food addiction [21–24]. The main findings of these studies imply that in some cases, the drive to eat palatable foods increases with these activated neuronal circuits, and these biological mechanisms may lead to addiction through learned experiences over a period of time.

Taste is a significant factor in food intake [25]. Although many other factors such as nutritional value, cost, and availability are also important in food intake, the deterministic effect of sensory appeal in food preference was reported [26]. In addition to a biological interaction between taste and addiction, behavioral outcomes were also observed in both human and animal models. In a rat model, postponed acetylcholine satiation response, greater sucrose intake, and increased dopamine secretion were reported after following a 10% sucrose solution and chow diet for 21 days [27]. Similar results were obtained in various studies, suggesting that in animal models, sugar bingeing could be related to addiction [28, 29]. Food addiction was also shown to have evolved from excessive intake of varied nutrients; thus, it may be subtyped [30]. Although human studies are very limited in this field of research, the evidence that sugar may exert even bigger effects than addictive drugs [31] makes the topic crucial in terms of public health and the health status of future generations.

The implication and translation of food addiction theory in humans is an important area of research. Despite the lack of a consensus opinion on diagnostic criteria for food addiction [32]. Gearhardt et al. suggested that food addiction be classed with drug addiction in the Diagnostic and Statistical Manual of Mental Disorders-IV (DSM-IV) [13]. The Yale Food Addiction Scale (YFAS), which is based upon DSM-IV, was developed to determine the presence of food addiction in those with at least three addiction symptoms for at least 1 year [33]. Basically, the YFAS quantifies the inability to limit consumption of specific foods despite repeated attempts, the nutritional behaviors that conflict with social and professional activities, and the degree of deprivation signs when specific foods were kept away [32].

To date, the YFAS has been validated in a non-clinical sample of children [34] and those obtaining weight-loss surgery [35] in French [36] and in Chinese [37]. Because cultural and socio-demographic features of populations vary largely, language validation of such tools is important for defining eating behaviors in various populations. Currently, a tool defining food addiction exists in Turkish [38]. However, this tool has been developed and validated on a smaller sample size with a limited age range. Therefore, the aim of this study was to test the Turkish version of the YFAS in a non-clinical and a larger sample that represents the public more extensively.

## Methods

### Participants and recruitment

A total of 1033 Turkish adults who were students or employees at Hacettepe University voluntarily participated in this study. The study sample consisted of 439 men and 594 women aged 19 to 65 years. Mean age was of 31.1 ± 11.78 years. The sample consisted of 41.8% were university students and 58.2% were employees; more than half (57.8%) graduated from high school, 30.9% graduated from college, and 11.3% graduated from secondary school or had less education. Students or employees in the Department of Nutrition and Dietetics were excluded from the study because their awareness of eating behavior might be different than in the general population. Pregnant or lactating women were also excluded because eating behaviors might change during that time of life. No other particular inclusion or exclusion criteria were used. The study protocol was approved by the Ethics Committee of Hacettepe University, and written informed consent was obtained from all participants.

Participants were recruited via email and announcements posted in several locations, such as the cafeteria, sport hall, library, and classrooms. An appointment was provided for each volunteer at the Nutrition Education and Research Unit in the Department of Nutrition and Dietetics. Researchers provided verbal and visual information on how to respond to items in each questionnaire. The participants spent about 30 min taking questionnaires (YFAS, Eating Attitudes Test-26 [EAT-26], and a demographics questionnaire). Reproducibility of the Turkish version of the YFAS was assessed by applying it twice within a 3-week interval to a sample of 1018 participants.

### Instruments

#### Yale Food Addiction Scale

The YFAS was developed by researchers at Yale University in 2009 to assess food addiction. The seven symptoms of substance dependence, assessed over the prior 12 months, using the DSM-IV diagnostic criteria [33], were modified. These symptoms were as follows: substance was taken in larger amounts and for longer periods than intended; repeated attempts to quit were unsuccessful; too much time was spent on eating and food; social, occupational, or recreational activities were foregone to eat; use continued despite knowledge of adverse consequences; tolerance to food was high; and withdrawal was suffered after not eating. Whether use of food caused clinically significant impairment was assessed separately. A continuous “symptom count,” indicating the number of dependence symptoms, and a dichotomous “diagnostic” score, indicating that an individual met the criteria for food addiction if he or she presented three or more symptoms and reported clinically significant impairment and/or distress were calculated. A total symptom count score was calculated for those meeting the criteria for the seven symptoms. Meeting the criteria for three or more of these domains was considered to indicate food addiction [33].

After the English version of the YFAS was obtained from researchers at Yale University (Dr. Ashley Gearhardt, Yale University, New Haven, USA), it was translated into Turkish by two bilingual researchers. Backward translation techniques were used to develop language-specific versions of the YFAS. Discrepancies between the original and translation were resolved by a professional English translator. The English and Turkish versions were given 3 weeks apart to a group of final year students from the Department of American Culture and Literature (*n* = 52), who had fluent English language skills. The total scores of both versions were similar to the total score from the original YFAS (*p* > 0.05) validating the tool.

#### Eating Attitudes Test-26

The EAT-26, widely used to measure symptoms of disordered eating of both the anorexic and bulimic variants, was developed by Garner et al. in 1982 [39]. It is based on the Eating Attitudes Test (EAT-40), validated in Turkish by Savasir and Erol in 1989 [40]. It includes 26 items in which the frequencies of attitudes and beliefs are rated using a 6-point scale. Participants scoring 20 or greater are considered at high risk for an eating disorder. In the current study, the Turkish version of the EAT-26, piloted by Baş et al. in 2004, was used. Evidence of its internal consistency reliability (Cronbach’s alpha = 0.70) and test–retest reliability over a 3-week period (0.98) was garnered in a community sample of Turkish adults [41]. EAT-26 was also administered along with the YFAS to test criterion-related validity.

#### Demographics questionnaire

Information about age, sex, health status, education, occupation, smoking habits, alcohol consumption pattern, and physical activity level were obtained.

### Data analysis

#### Internal structure

The factorial structure of the Turkish version of the YFAS was examined by exploratory factor analysis, conducted using IBM SPSS Statistics Version 21. The principal component factor analysis with varimax rotation was conducted. This means that the final component will be at right angles with each other. With this conversion, we can assume that the information explained by one component is independent of the information in the other component. Data suitability for exploratory factor analysis was assessed using the Kaiser–Meyer–Olkin measure of sampling adequacy and Bartlett’s test of sphericity. Criteria for factor loadings included item values ≥ 0.40 on the primary factor and values no more than ≤ 0.20 on other factors. The model obtained in the exploratory factor analysis was tested on the study sample (*n* = 1033) using confirmatory factor analysis. The chi-square test (*χ*^2^), comparative fit index (CFI), normed fit index (NFI), goodness of fit index (GFI), and root mean square error of approximation (RMSEA) were calculated using LISREL version 8.7 [42–45].

#### Reliability

Descriptive statistics of the total score and eight item subscale scores for the YFAS were calculated. Internal consistency reliability of the Turkish version of the YFAS was tested using Cronbach’s alpha. The Pearson product-moment correlation analysis was used to establish its temporal stability, allowing test–retest reliability to be explored. The criterion-related validity of the total YFAS score and total EAT-26 score was examined by the chi-square distribution of participants with normal/abnormal eating behaviors and food addiction/none-food addiction [44, 45].

## Results

General characteristics of the study sample are given in Table 1. The mean age of participants was 31.07 ± 11.77 years with a range of 19–65 years. Majority of the participants (88.6%) had high school or university diploma. Non-smoker (79.3%), non-alcohol consumer (78.2%), and non-exercisers (73.7%) comprised the majority of the study population. The mean BMI of participants was 24.73 ± 5.05 kg/m^2^, and respectively 28.4% and 14.7% of the study population were overweight and obese (Table 1).

**Table 1:** General characteristics of the participants

Variable *n* (%)		Male (*n* = 439)	Female (*n* = 594)	Total (*n* = 1033)
Age (years)	19–25	207 (47.2)	313 (52.7)	520 (50.3)
	25–35	106 (24.1)	101 (17.0)	207 (20.0)
	35–45	47 (10.7)	85 (14.3)	132 (12.8)
	45–55	53 (12.1)	74 (12.5)	127 (12.4)
	55–65	26 (5.9)	21 (3.5)	47 (4.5)
Education	No schooling to primary school	12 (2.7)	68 (11.4)	80 (7.8)
	Secondary school	16 (3.6)	21 (3.5)	37 (3.6)
	High school	237 (54.0)	360 (60.6)	597 (57.8)
	University/postgraduate	174 (39.7)	145 (24.4)	319 (30.8)
Occupation	No work, staying at home	– (0.0)	132 (22.2)	132 (12.8)
	Student	153 (34.9)	279 (47.0)	432 (41.8)
	Laborer/officer	158 (36.0)	103 (17.3)	261 (25.3)
	Self-employment	47 (10.7)	19 (3.2)	66 (6.4)
	Retired	38 (8.7)	12 (2.0)	50 (4.8)
	Other	43 (9.7)	49 (8.3)	92 (8.9)
Smoking status	Non-smoker	318 (72.4)	501 (84.3)	819 (79.3)
	Smoker	121 (27.6)	93 (15.7)	214 (20.7)
Alcohol consumption status	Non-consumer	308 (70.2)	500 (84.2)	808 (78.2)
	Regular consumers	131 (29.8)	94 (15.8)	225 (21.8)
Physical activity	Non-exercisers	306 (69.7)	455 (76.6)	761 (73.7)
	Regular exercisers	133 (30.3)	139 (23.4)	272 (26.3)
BMI (kg/m^2^)	Underweight	24 (5.5)	73 (12.3)	97 (9.4)
	Normal	183 (41.7)	308 (51.9)	491 (47.5)
	Overweight	174 (39.6)	119 (20.0)	293 (28.4)
	Obese	58 (13.2)	94 (15.8)	152 (14.7)

### Factor analysis of YFAS items

The Kaiser–Meyer Olkin statistics was 0.909, showing that sample adequacy was high enough for factor analysis. Bartlett’s test (*χ*^2^ value) of sphericity was 8540.98 (*p* < 0.001), indicating the data was suitable for exploratory factor analysis. All factors with eigenvalues greater than 1 were identified.

Factor analysis was performed on seven YFAS items using principal component factor analysis with varimax rotation. The items were substance taken in larger amounts and for longer periods than intended; repeated unsuccessful attempts to quit; too much time spent on eating and food; giving up social, occupational, or recreational activities to eat; using continues despite knowledge of adverse consequences to eating behaviors; tolerance to food; and withdrawal from not eating. As shown in Table 2, all the primary factor loadings were at least 0.45, and no items cross-loaded onto other factors. Factor loadings ranged between 0.45 and 0.79 (Table 2). The analysis revealed that together, the seven factors explain 69.8% of the variance in the scale items.

Confirmatory factor analysis on the entire sample (*n* = 1033) tested the model obtained in the exploratory factor analysis. Table 2 presents the standardized coefficients as well as the descriptive statistics obtained for the total sample. Fit indices indicated that eight items of the YFAS represented the item responses in Turkish adults: S–B *χ*^2^ = 676.27, df = 168, *p* < 0.001, CFI = 0.950, RMSEA = 0.054, NFI = 0.94, and GFI = 0.98. Each item loaded significantly on the specified factor (*p* < 0.001 for each).

### Internal consistency

Descriptive statistics (means and standard deviations) and internal consistency values of the YFAS items are given in Table 2.

### Reliability

Results showed that the YFAS subscales have high test–retest reliabilities over a 3-week period (*n* = 1018, statistical power = 0.813). The test–retest reliability among all participants was 0.77 for substance taken in larger amounts and for longer periods than intended; 0.52 for repeated unsuccessful attempts to quit; 0.65 for too much time spent on eating and food; 0.82 for giving up social, occupational, or recreational activities to eat; 0.46 for tolerance to food; 0.76 for withdrawal from not eating; and 0.69 for using causes clinically significant impairment. The reliability for using continues despite knowledge of adverse consequences to eating behaviors was not calculated because it had only one component (Table 2).

### Criterion-related validity

The Pearson product-moment correlation coefficients were calculated for the YFAS scores and EAT-26 scores for all participants to test criterion reliability (*n* = 1033). The bivariate and partial correlation coefficients between total YFAS score and total EAT-26 score for diagnosis in the entire study population were 0.230 (*p* < 0.001). It was 0.189 for the number of symptoms (*p* < 0.001). The chi-square distribution of participants with normal/abnormal eating behaviors diagnosed by EAT-26 and food addiction/none-food addiction is given at Table 3. It was shown that 41.8% of the participants with food addiction also had eating disorder, whereas only 11.1% of the participants without food addiction had eating disorder (chi-square = 80.892, *p* = 0.001).

**Table 2:** Descriptive statistics, and exploratory and confirmatory factor analyses of the YFAS

Items	Total Sample (*n* = 1033)	EFA sample 1 (*n* = 1033)	CFA sample 2 (*n* = 1033)	Cronbach’s alpha coefficients
	*X* ± SD or %	Factor loadings	Standardized coefficients	
Substance taken in larger amounts and for longer period than intended				.769
1. I find that when I start eating certain foods, I end up eating much more than planned	1.8 ± 1.27	.756	.81	
2. I find myself continuing to consume certain foods even though I am no longer hungry	1.7 ± 1.32	.768	.89	
3. I eat to the point where I feel physically ill	1.1 ± 1.23	.656	.83	
Repeated unsuccessful attempts to quit				.516
4. Not eating certain types of food or cutting down on certain types of food is something I worry about.	1.4 ± 1.41	.452	.66	
22. I want to cut down or stop eating certain kinds of food.	54.7	.744	.74	
24. I have been successful at cutting down or not eating these kinds of food.	39.8	.699	.78	
25. How many times in the past year did you try to cut down or stop eating certain foods altogether?	1.9 ± 1.8	.652	.57	
Too much time spent on eating and food				.651
5. I spend a lot of time feeling sluggish or fatigued from overeating	1.3 ± 1.32	.583	.79	
6. I find myself constantly eating certain foods throughout the day	1.1 ± 1.25	.638	.89	
7. I find that when certain foods are not available, I will go out of my way to obtain them. For example, I will drive to the store to purchase certain foods even though I have other options available to me at home.	1.0 ± 1.19	.520	.77	
Giving up social, occupational or recreational activities to eat				.820
8. There have been times when I consumed certain foods so often or in such large quantities that I started to eat food instead of working, spending time with my family or friends, or engaging in other important activities or recreational activities I enjoy.	0.5 ± 0.89	.636	.96	
9. There have been times when I consumed certain foods so often or in such large quantities that I spent time dealing with negative feelings from overeating instead of working, spending time with my family or friends, or engaging in other important activities or recreational activities I enjoy.	0.4 ± 0.89	.721	1.00	
10. There have been times when I avoided professional or social situations where certain foods were available, because I was afraid I would overeat.	0.4 ± 0.89	.787	.93	
11. There have been times when I avoided professional or social situations because I was not able to consume certain foods there.	0.4 ± 0.81	.781	.95	
Using continues despite knowledge of adverse consequences to eating behaviors				–
19. I kept consuming the same types of food or the same amount of food even though I was having emotional and/or physical problems.	29.0	.663	–	
Tolerance to food				.464
20. Over time, I have found that I need to eat more and more to get the feeling I want, such as reduced negative emotions or increased pleasure.	31.6	.599	.95	
21. I have found that eating the same amount of food does not reduce my negative emotions or increase pleasurable feelings the way it used to.	44.1	.772	.90	
Withdrawal from not eating				.760
12. I have had withdrawal symptoms such as agitation, anxiety, or other physical symptoms when I cut down or stopped eating certain foods.	0.5 ± 0.95	.645	.95	
13. I have consumed certain foods to prevent feelings of anxiety, agitation, or other physical symptoms that were developing.	0.8 ± 1.06	.481	.88	
14. I have found that I have elevated desire for or urges to consume certain foods when I cut down or stop eating them.	1.0 ± 1.12	.448	.84	
Using causes clinically significant impairment				.688
15. My behavior with respect to food and eating causes significant distress	0.7 ± 1.11	.532	.96	
16. I experience significant problems in my ability to function effectively (daily routine, job/school, social activities, family activities, health difficulties) because of food and eating	0.5 ± 0.93	.621	.95

**Table 3:** Presence of food addiction and eating disorder in the study population [*n*(%)]

		Food addition			
		Yes	No	Total	*p*
Eating disorder	Yes	51 (41.8%)	101 (11.1%)	152 (14.7%)	0.001
	No	71 (58.2%)	810 (88.9%)	881 (85.3%)	
	Total	122 (100.0%)	911 (100.0%)	1033 (100.0%)	

Chi-square = 80.892, *p* = 0.001

## Discussion

In this study, we investigated the psychometric features of the Turkish version of the YFAS in a non-clinical sample of adults. This scale had moderately good internal consistency and construct validity in its diagnosis and symptom count. As with the original version, nearly all items were significantly correlated with their total score. Hence, it can be suggested that the Turkish version of the YFAS is a useful tool in a non-clinical setting for defining food addiction.

Despite good internal consistency and construct validity, a few items (item 4: not eating certain types of food or cutting down on certain types of food is something I worry about; Item13: I have consumed certain foods to prevent feelings of anxiety, agitation, or other physical symptoms that were developing; item 14: I have found that I have elevated desire for or urges to consume certain foods when I cut down or stop eating them) exhibited relatively lower exploratory factors than other items. Of these, the first assesses the food addiction criterion named “repeated unsuccessful attempts to quit.” In other versions of the YFAS, item 22 (I want to cut down or stop eating certain kinds of food), item 24 (I have been successful at cutting down or not eating these kinds of food), and item 25 (How many times in the past year did you try to cut down or stop eating certain foods altogether?) which were also included in the criterion “repeated unsuccessful attempts to quit” had low factor loadings [36, 37, 46]. Although we showed that these three items had more powerful outcomes than item 4, it appears that under the criterion “repeated unsuccessful attempts to quit,” they may have slightly lower sensitivity when distinguishing food addiction [46]. Because previous studies suggested reserve scoring these items, we also reserved item 4. In addition, “repeated unsuccessful attempts to quit” can be a repetitive attitude in constituting healthy eating habits or weight loss attempts [46]. Moreover, “worry about not eating certain types of food or cutting down on certain types of food” might not be a familiar concept in Turkish eating culture. Therefore, future studies may focus on developing and improving this criterion in the Turkish version of the YFAS.

Unlike other validation studies, current study exhibited a relatively moderate Cronbach’s alpha for the criterion “tolerance to food” although all factor analysis for item 20 (over time, I have found that I need to eat more and more to get the feeling I want, such as reduced negative emotions or increased pleasure) and item 21 (I have found that eating the same amount of food does not reduce my negative emotions or increase pleasurable feelings the way it used to) showed strong correlations. Similarly, item 13 (I have consumed certain foods to prevent feelings of anxiety, agitation, or other physical symptoms that were developing) and item 14 (I have found that I have elevated desire for or urges to consume certain foods when I cut down or stop eating them) exhibited relatively moderate explanatory factors. Because our level of significance was not observed in other language validation studies for the “tolerance to food” criterion and items 13 and 14, these can be attributable to socio-cultural and socio-economic differences in the Turkish population. The influence of cross-cultural or host culture experiences on eating behaviors and nutritional status is well documented [47, 48]. In addition, our study design relied on self-reporting in all cases. Hence, nutrition-related cultural differences or possible deviations related to subject differences may have contributed to these outcomes.

A slight positive correlation was obtained between EAT-26 and the Turkish version of the YFAS in this study. However, the frequency of food addiction was significantly higher than the frequency of eating disorder as expected. This was based on the distinct structures of eating behaviors that these tools examine or sample differences. The EAT-26 measures mainly symptoms and concerns of eating disorders, whereas YFAS measures mainly symptoms of addicted behavior. Moreover, other language validation studies exhibited high correlations between YFAS and EAT-26 or other eating behavior scales, but not all of these scales appeared to be consistently correlated with YFAS [37].

The prevalence of food addiction was 11.8% in this study sample. This was comparable with that seen in other studies conducted in the USA (11.4%) [11], Germany (8.8%) [12], and France (8.7%) [36]. These outcomes suggest that the Turkish version of the YFAS exerts similar psychometric features to previously validated versions in non-clinical settings. In addition, the heterogeneous structure of the study sample in terms of age, sex, body mass index, and socioeconomic characteristics made the tool useful for different groups.

In conclusion, we demonstrated that the Turkish version of the YFAS is an efficient tool that can be used in food addiction investigations. Because a non-clinical sample was used in this study, subsequent studies may consider assessing clinical subjects to better elucidate the psychopathologic and psychiatric factors associated with food addiction.

## Data Availability

The datasets used and/or analyzed during the current study are available from the corresponding author on reasonable request.

## Abbreviations

CFI: Comparative fit index
DSM-IV: Diagnostic and Statistical Manual of Mental Disorders-IV
EAT-26: Eating Attitude Test-26
GFI: Goodness of fit index
NFI: Normed fit index
RMSEA: Root mean square error of approximation
YFAS: Yale Food Addiction Scale
